# Multi-Substance Use Behaviors: Prevalence and Correlates of Alcohol, Tobacco and Other Drug (ATOD) Use among University Students in Finland

**DOI:** 10.3390/ijerph18126426

**Published:** 2021-06-14

**Authors:** Walid El Ansari, Abdul Salam

**Affiliations:** 1 Department of Surgery, Hamad General Hospital, Hamad Medical Corporation, Doha 3050, Qatar; 2 College of Medicine, Qatar University, Doha 3050, Qatar; 3 School of Health and Education, University of Skovde, 541 28 Skövde, Sweden; 4 Department of Epidemiology and Biostatistics, King Fahad Specialist Hospital, Dammam 31444, Saudi Arabia; abdul.salam@kfsh.med.sa

**Keywords:** alcohol, tobacco, illicit/other drug, ban, policy, education, Finland, university students

## Abstract

Virtually no studies appraised the co-use of alcohol, tobacco, and other drug (ATOD) among Finn undergraduates. We assessed the associations between sociodemographic, health, academic, policy, and lifestyle characteristics (independent variables); and individual, multiple and increasing ATOD use (dependent variables) using regression analyses. Data were collected by online questionnaire at the University of Turku, Finland (1177 students). Roughly 22% of the sample smoked, 21% ever used illicit drug/s, 41% were high frequency drinkers, and 31.4%, 16.3%, and 6.7% reported 1, 2, or 3 ATOD behaviors respectively. Individual ATOD use was significantly positively associated with the use of the other two substances [adjusted odds ratio (Adj OR range 1.893–3.311)]. Multiple ATOD use was negatively associated with being single (*p* = 0.021) or agreeing with total smoking or alcohol ban policy on campus (*p* < 0.0001 for each); but positively associated with not living with parents (*p* = 0.004). Increasing ATOD behaviors were significantly less likely among those agreeing with total smoking or alcohol ban policy on campus (*p* range 0.024 to <0.0001). Demographics significant to either individual, multiple, or increasing ATOD use included males, being single, not living with their parents during semesters, and to some extent, religiosity. Age, depressive symptoms, perceived stress, self-rated health, health awareness, income sufficiency, and academic variables were not associated with individual, multiple, or increasing ATOD use. Education and prevention efforts need to reinforce abstinence from ATOD, highlight their harmful outcomes, and target risk groups highlighted above. University strategies should be part of the wider country-wide successful ATOD control policies.

## 1. Introduction

Alcohol, tobacco, and other drug (ATOD) use is a common societal problem globally. Worldwide, young people (15–24 years) remain more likely to experience substance misuse compared to the general population [1,2]. Young adulthood is a peak time for experimentation with substances, and the college environment is inherently risky for substance use behaviors [3]. Among college students, the prevalence for ATOD use ranged between 41.3–69.8% [4,5,6]. A study of young adult past-month cigarette smokers revealed that 53% also reported past month cannabis use [7], and 47.9% of polysubstance-using students reported consuming tobacco during their last cannabis use [8].

There is substantial correlation between the use of tobacco, alcohol, and other substances [6]. Cannabis use might predict transitions into, and maintenance of, tobacco use [9]. Evidence supports that marijuana and cigars are strongly associated and use of one substance predicts use of the other [10]. Each individual component of ATOD is a recognized health risk with undesirable consequences [11]. Hence, the simultaneous use of ATOD could lead to serious consequences, where co-use of alcohol and marijuana was associated with higher rates of marijuana and alcohol use disorders and negatively influenced treatment outcomes for both substances [12].

A number of variables are linked to ATOD use. A review found that risk factors of cannabis use included being male, tobacco smoking, and alcohol use [13]. An examination of the association between mental health problems/difficulties and ATOD use among university students found that higher cigarette and drug use were significantly associated with higher scores of a screening scale for severe mental illness [14]. Likewise, among students, stress was indirectly related to both alcohol and marijuana [15]. Similarly, initiation of ATOD (including cigarette) use at early age predicted ATOD misuse [16]. Furthermore, 15.4% of students had financial problems as a negative consequence of substance misuse [6]; and research found that student loans have negative effects on young people in terms of smoking, and heavy drinking [17]. As students involve in risky lifestyles (smoking, alcohol, and substance misuse), such actions bear negatively on their health and also on their education and academic achievement [18,19].

To the best of our knowledge, no research has examined the collective ATOD behaviors among university students in Finland. For these young Finn adults, the very few studies undertaken assessed the components of ATOD individually, e.g., illicit drug use alone [20], tobacco use alone [21], or alcohol consumption alone [22]. Cannabis use has increased in Finland despite the strict policy [23]. 

Therefore, the current study, conducted at the University of Turku in Finland, assessed the relationships between 2 sociodemographic (gender, age), 2 mental health variables (depressive symptoms, perceived stress), and 2 policy variables (agreement with smoking and alcohol bans at university) as independent variables; and 3 ATOD risk factors (smoking, frequency of alcohol consumption, and illicit drug/s use) as dependent variables. The specific objectives were to: describe the prevalence of individual and multiple ATOD risk factors; examine the correlates of individual and multiple ATOD risk factors; and, assess the correlates of increasing ATOD risk factors. To the best of our knowledge, the current study is the first to assess such factors and the relationships among undergraduate university students in Finland.

## 2. Materials and Methods 

### 2.1. Ethics, Sample, and Data Collection

The University Research and Ethics Committee approved the study (# Lausunto 10/2010). During the academic year 2013–2014, all undergraduates at all faculties of the University in Turku were sent initial invitation emails to partake in the online English survey. The study is fully described elsewhere [20]. Two weekdays after the initial email invitation of students, a follow up reminder email was sent again to all undergraduates. In addition, three posters about the study were exhibited at the students’ cafeteria at the University, and a reminder was announced on the University intraweb. Participation was voluntary and anonymous, and students were informed that by completing the online survey, they consent to participate in the study. As students completed the online survey and ‘submitted’ their completed questionnaires, their responses were saved and directed to the Student Management Office at the University. This Office gathered the online responses, and data were electronically entered into an excel sheet ensuring high quality assurance. After completion of this phase, the data was sent to the research team who electronically imported the data (no identifiers) into SPSS for analysis. Data were confidential and protected at all times. The number of students invited was 4387; 1177 completed questionnaires were received. Average respondent age was 22.96 ± 5.21 years; females comprised 70.4%. Response rate was≈27%. The University of Turku is the third largest university in Finland, comprising faculties of Education, Humanities, Law, Medicine, Science and Engineering, Social Sciences, and Economics. Smoking is permitted only in designated areas.

### 2.2. Health and Wellbeing Questionnaire 

The self-administered questionnaire gathered general health data: socio-demographic (sex, age, year of study, living arrangements during university terms); health (self-rated general health, health awareness); lifestyle [frequency of alcohol consumption, illicit drug use (IDU), smoking]; mental wellbeing variables (depressive symptoms, perceived stress), university related educational questions (academic achievement compared to peers), and information on religiosity and income sufficiency. The tool was used and field-tested across many student populations [11,24,25,26]. Variables with several response options were later dichotomized as shown in Table 1.

#### 2.2.1. Sociodemographic Variables

Age, sex, and year of study at university were based on self-reports. Age was used as a continuous variable.

Marital status: “What is your marital status?” Response options included single, married, or other (please specify), dichotomized into ‘single’ vs. ‘married or in relationship’ [24,25].

Accommodation (living arrangements) during semester: “Where do you live during university/college term time?”, dichotomized into ‘living with parents’ vs. ‘not living with parents’ [25].

Religiosity (personal importance of religious faith): the extent to which participants agreed/disagreed with the statement: “My religion is very important for my life”, 1 = ‘strongly agree’, 2 = ‘somewhat agree’, 3 = ‘neither agree nor disagree’, 4 = ‘somewhat disagree’, and 5 = ‘strongly disagree’, recoded into 2 categories based on agreement/disagreement (1, 2, 3 = 1 vs. 4, 5 = 2) [24,25].

Income sufficiency: “How sufficient do you consider your income?” with four Likert scale responses (“always sufficient”, “mostly sufficient”, “mostly insufficient”, or “insufficient”) which were then dichotomized into “Mostly or always sufficient” vs. “other” [20].

#### 2.2.2. ATOD Use and Policy Variables

Smoking (1 item): students were asked “Within the last three months, how often did you smoke (cigarettes, pipe, cigarillos, cigars)?” (three response scales: ‘daily’, ‘occasionally’, ‘never’). We dichotomized it into two categories: ‘daily/occasionally’ vs ‘never’ [21,27].

Frequency of alcohol consumption (1 item): “Over the past 3 months how often did you drink alcohol, for example, beer?” (6 response options: ‘never’, ‘once a week or less’, ‘once a week’, ‘a few times each week’, ‘every day’, and ‘a few times each day’), later dichotomized into low frequency = ‘never’ or ‘once a week or less’, or high frequency = ‘once a week’, or ‘a few times each week’, or ‘every day’, or ‘a few times each day’ [18,22].

Illicit drug/s use (1 item): ‘‘Have you ever use/used drugs?’’ (‘‘yes, regularly’’, ‘‘yes, but only a few times’’, ‘‘never’’) e.g., marijuana, cocaine, heroin, crack, LSD, amphetamines. As the distribution of this variable was highly skewed, we dichotomized it into two categories: Ever = ‘‘yes regularly or yes but only a few times’’, and Never = ‘‘Never used’’ [25].

Total smoking ban (1 item): To what extent do you agree with the statement? “There should be no smoking on university premises at all’’ (five-point scale: ‘strongly disagree’, ‘disagree’, ‘neutral’, ‘agree’, ‘strongly agree’). We dichotomized it into two categories: ‘Strongly disagree/Disagree’ vs ‘Strongly agree/Agree’ [28].

Total alcohol ban (1 item): To what extent do you agree with the statement? “Alcohol should not be sold at the university” (five-point scale: ‘strongly disagree’, ‘disagree’, ‘neutral’, ‘agree’, ‘strongly agree’). We dichotomized it into two categories: ‘Strongly disagree/Disagree’ vs ‘Strongly agree/Agree’ [11].

#### 2.2.3. Health Variables 

Self-rated general health: “How would you describe your general health?” (1 = ‘poor’, 2 = ‘fair’, 3 =’good’, 4 =’very good’, and 5 = ‘excellent’), dichotomized into ‘poor/fair’ vs. ‘good/very good/excellent’ adopted from [29].

Health awareness: “To what extent do you keep an eye on your health?” (1 = ‘not at all’, 2 = ‘not much’, 3 =’to some extent’, and 4 = ‘very much’), dichotomized into ‘not at all/not much’ vs. ‘to some extent/very much’ [20]. 

Depressive symptoms (20 items): using the Modified Beck Depression Inventory (M-BDI) [30,31]. Sample items included: “I feel sad,” “I feel I am being punished,” “I have thoughts of killing myself,” “I have lost interest in other people,” “I have to force myself to do anything”. BDI computes a single score for individual respondents by summing their responses for all items of the scale. Higher scores indicate more depressive symptoms.

Perceived Stress Scale (4 Items): Cohen’s Perceived Stress Scale (PSS) in its four-item short form [32] assessed the extent to which participants considered life situations to be stressful. PSS−4 measures the degree to which situations in one’s life over the past month are appraised as stressful. The questions detect how unpredictable, uncontrollable, and overloaded respondents find their lives. All items began with: “In the past month, how often have you felt...?” (5 point scale: 1 = ‘never’, 2 = ‘almost never’, 3 = ‘sometimes’, 4 = ‘fairly often’, 5 = ‘very often’). In our sample, Cronbach’s alpha of PSS was 0.75. A perceived stress score was generated by summing the responses to the 4 questions, where higher scores indicate more perceived stress [22].

#### 2.2.4. Academic Variables 

We assessed academic performance using 2 items:

Students’ internal reflection on their academic performance (importance attached to achieving good grades): “How important is it for you to have good grades at university?” (4 response categories, 1 = ‘very important’, 2 = ‘somewhat important’, 3 = ‘not very important’, and 4 = ‘not at all important’), dichotomized into 1 = ‘somewhat important or very important’ vs 2 = ‘other’ [20].

Students’ subjective comparative appraisal of their performance in comparison with their peers: “How do you rate your performance in comparison with your fellow students?” 1 = ‘much better’, 2 = ‘better’, 3 = ‘same’, 4 = ‘worse’, 5 = ‘much worse’, dichotomized based on perceived better performance (4, 5 = 1 vs 1, 2, 3 = 2) [20].

### 2.3. Statistical Analysis

Descriptive and inferential statistics characterized the study sample and tested hypotheses. Quantitative variables are presented as mean ± standard deviation, while numbers (percentage) were used for qualitative variables. As ATOD (alcohol use, smoking, and illicit drug/s use) behaviors were different for males and females, descriptive analysis of the variables was undertaken separately for gender. Independent sample t-test was used to compare all the quantitative variables (age, depressive symptoms, and perceived stress), while Pearson Chi-Square test was used for all the qualitative variables (e.g., marital status, total alcohol ban, and total smoking ban etc.) between male and female. 

Separate multiple binary logistic regression models (smoking, alcohol use, and IDU) were analyzed to assess the association between gender, age, marital status, accommodation during semester, health awareness, self-rated general health, religiosity (importance of religion in life), income sufficiency, academic performance compared to peers, importance of achieving good grades, depressive symptoms, perceived stress, total alcohol ban, total smoking ban and ATOD behaviors. All two-way gender interactions were assessed but were not statistically significant. Adjusted odds ratio (AOR) and 95% confidence intervals for the AOR were reported. 

A level of multiple ATOD risk factors (0–3) variables was created based on the responses for alcohol use, smoking, and IDU, where 0 indicated no risk behavior at all, while 3 indicated all the three risk behaviors. We used multiple linear regression analysis to assess the association between gender, age, marital status, accommodation during semester, health awareness, self-rated general health, religiosity, income sufficiency, academic performance compared to peers, importance of achieving good grades, depressive symptoms, perceived stress, total alcohol ban, total smoking ban and level of multiple ATOD risk behaviors. Regression coefficient with their 95% confidence inter were reported.

We also ran three separate multiple binary logistic regression models (1 ATOD risk behavior versus No risk behavior, 2 ATOD risk behaviors versus No risk behavior, and 3 ATOD risk behaviors versus No risk behavior) to assess the association between increasing ATOD risk behaviors and gender, age, marital status, accommodation during semester, health awareness, self-rated general health, religiosity, income sufficiency, academic performance compared to peers, importance of achieving good grades, depressive symptoms, perceived stress, total alcohol ban, and total smoking ban. Adjusted odds ratio (AOR) and 95% confidence interval for the AOR were reported. Statistical significance was set at “*p*” < 0.05 (two-tailed). Hosmer-Lemeshow assessed the model’s Goodness-of-fit. The Statistical Package for Social Sciences Version 25 (SPSS) was used.

## 3. Results

### 3.1. Characteristics of the Sample

The sample comprised 1177 undergraduates of which about 50% were in their second (29.4%) and third (29.4%) year of study at university. All the faculties of the university were represented, where roughly half the respondents were studying the disciplines Technology and Science (28.5%) or Humanities (28.5), with less students from the disciplines of Education and Law (16.4%), Medicine (14.6%), and Economics (12%) (data not presented).

Table 1 shows that that the mean age of respondents was 22.96 ± 5.21 years, 70% were females, about half were single, more than half (66.3%) were not living with their parents during the university semesters, and 58% felt that their monthly income was always or mostly insufficient. The majority of these undergraduates reported high health awareness (86.4%) and self-rated general health as good/very good/excellent (92.6%). The sample’s mean BDI and perceived stress scores were 50.88 ±18.4 and 14.14 ± 3.20 respectively. The gender differences across these variables are depicted in the table. 

### 3.2. Prevalence of Individual and Multiple ATOD Risk Factors

Table 1 also shows that for ATOD features, one fifth of the sample (22%) smoked daily/occasionally or ever used illicit drug/s (21%), while 41% reported high frequency of drinking alcohol during the last 3 months. Less than half the undergraduates reported no ATOD risk factor, while the remaining respondents registered 1, 2 or 3 ATOD risk factors (31.4%, 16.3%, and 6.7% respectively). The gender differences across these variables are depicted in the table. Illicit drugs reported by the sample included cannabis, marijuana, LSD, amphetamines (MDMA), dextromethorphan (DXM), gamma hydroxybutyrate (GHB), various opioids, psilocybin, hallucinogenic mushrooms, codeine, ecstasy, cocaine, LSD, ketamine, subutex, nitros, ephedrine, benzodiazepine, poppers, modified drugs, design drugs, and psychedelics. 

### 3.3. Correlates of Individual ATOD Risk Factors

Table 2 depicts the correlates of each of the three ATOD use variables. For daily/occasional smoking, the only significant predictors were having mostly/always sufficient monthly income, agreement with a no smoking policy on university premises, high frequency drinking, and ever IDU. In addition, not living with parents during university semesters displayed borderline significance (*p* = 0.052) for daily/occasional smoking.

In terms of high frequency drinking the only significant predictors were agreement with total alcohol ban policy on university premises policy, daily/occasional smoking and ever illicit drug use. In addition, higher health awareness displayed borderline significance (*p* = 0.054) for high frequency drinking. As for ever IDU, the only significant predictors were being male, single, not living with parents during university semester times, reporting positive self-rated general health, high depressive symptoms, agreeing with a total smoking ban policy on university premises, and being a daily/occasional smoker or a high frequency drinker.

### 3.4. Correlates of Multiple ATOD Use

Table 3 shows the correlates of multiple ATOD use. Single students were significantly less likely to report multiple ATOD behaviors, while those not living with parents during university semesters were significantly more likely to report multiple ATOD behaviors. In addition, respondents in agreement with total smoking or alcohol ban policy on university premises were significantly less likely to exhibit multiple ATOD behaviors. All the remaining variables were not associated with multiple ATOD behaviors.

### 3.5. Correlates of Increasing ATOD Use

Table 4 depicts the correlates of increasing ATOD use. Three variables were significantly associated with all three levels of ATOD use. For instance, students in agreement with total smoking or alcohol ban policy on university premises were significantly less likely to be engaged in all three levels of ATOD behaviors. Conversely, not living with parents during university semesters was significantly and positively associated with all the three levels of ATOD behaviors, reaching a striking level of 8.5 times in the case of 3 ATOD behaviors. Low importance of religion in one’s life was significantly positively associated with two or three ATOD behaviors. In addition, two variables (gender and marital status) were significantly associated with reporting two ATOD behaviors, where males were more likely and singles were less likely to display two ATOD behaviors.

## 4. Discussion

Risky behaviors and use of different substances seem to congregate among the same people [33,34], where among young persons in Canada and Europe, smoking and heavy drinking were associated with problematical cannabis use [35,36]. Multi-substance use carries added risks compared to single-substance use, e.g., higher likelihood of use disorders, increased psychosocial problems, and inferior cessation outcomes [37]. Despite this, virtually no data exists on the co-occurrence of multiple ATOD behaviors among Finn undergraduates. The present study is the first to bridge this knowledge gap to assess the prevalence and correlates of individual, multiple and increasing ATOD risk factors among this important young adult population. Although that the etiology of substance use is quite comparable across different substances, appraising the variables that predict uniquely across different substances assists to recognize prominent and promising foci for potential interventions [38].

As for the prevalence of individual ATOD use, the prevalence we observed for current smoking, ever use of illicit drug/s, and high frequency drinking agree with other research. Such smoking prevalence agrees with UK students across 7 universities that employed the same tools as the current study, where 27.8% smoked [28]. The observed smoking prevalence among the current Finns is a concern, particularly that in 2019, 18.5% of young people attending vocational institutes smoked [39]. Likewise, our observed IDU is comparable with the USA, where 20% of college students used marijuana [40], although our rate was less than rates of students in Italy (50.4%) or Switzerland (44%) [41,42]. Equally, the current high frequency drinking rate concurs with the 42.7% of undergraduates in England, Wales, and Northern Ireland [24]; and with students’ alcohol consumption in Finland, where 33% were risky drinkers [43].

In terms of the correlates of individual ATOD behaviors, in the current study, the most prominent correlates of the use of any of the three substances were the use of the other two substances. Indeed, use of any one substance was associated with the use of the other two, and the associations were positive, significant (*p* range: 0.028 to < 0.0001), and considerable, stretching from 1.893 (likelihood of ever illicit drug users reporting high frequency drinking) to 3.311 (likelihood of smokers ever using an illicit drug). Such relationships between multi-substance use concur with the positive relationships between one substance use and the other in e.g., Germany and USA [44,45]. Likewise, in the UK, regular IDU was significantly more likely among students with heavy episodic drinking or possible alcohol dependency [25], further highlighting the connections between substance misuse, alcohol, and tobacco [6]. Our findings support the gateway drug supposition, where legal substance use (tobacco, alcohol, and marijuana in some countries) can lead to illegal substance misuse. 

Regarding the rates of multiple (2 or 3) ATOD behaviors, such multi-ATOD behaviors require attention, particularly given that health-endangering activities, e.g., smoking, alcohol and IDU often cluster [26,46]. We found that multiple ATOD behaviors were significantly negatively associated with being single or agreeing with total smoking or alcohol ban on campus, and significantly positively associated with not living with parents. Such findings support other research, where higher agreement with smoking ban on campus was significantly negatively associated with higher levels of multiple ATOD use [11].

As for increasing ATOD use, again Finnish students in agreement with total smoking or alcohol ban on campus were significantly less likely to report increasing ATOD use levels (*p* 0.024 to <0.0001). This is similar to research elsewhere, where agreement with campus total smoking ban was negatively associated with increasing ATOD behaviors [11]. However, while we found that agreement with alcohol ban policy on campus was significantly less likely to be associated with *all* levels of increasing ATOD use, other research [11] reported that agreement with alcohol ban policy was significantly negatively associated with only *one* level of increasing ATOD use. Such discrepancy might be due to the difference in prevalence of high frequency drinking, being 3.8% [11] as opposed to the current study where it was about tenfold, hence exerting a significant contribution in the regression analysis of increasing ATOD use.

The current study observed a set of demographic variables that were related to either individual, multiple, or increasing ATOD use. For instance, males had significantly higher odds of increasing levels of ATOD use, agreeing with research where male undergraduates were more likely to smoke, use illicit drug/s, and report high frequency drinking [11]. Likewise, being single was protective to ever IDU, negatively associated with multiple ATOD use, and protective to some (but not all) levels of increasing ATOD use, supporting that not being married was protective against substance use [47]. Similarly, in the current sample, students not living with their parents during the semesters were significantly more likely to report ever IDU, and multiple and increasing levels of ATOD behaviors (Adj OR range: 1.761 to 8.536). These findings support research among universities in the UK, where undergraduates living with parents displayed significantly lower odds across several alcohol consumption indicators [24]. We also observed that importance of religion was not associated with individual ATOD use, was of border significance for multiple ATOD use, but was significantly inversely associated with increasing (2 or 3) ATOD behaviors, where students with lower religiosity were more likely to be associated with increasing (2 or 3) ATOD behaviors. In support, others found that rituals (most notably, prayer), along with exposure to religious environments and institutions in the real world influence self-control on the scale of weeks, months, and years [48]; and likewise, a recent review concluded that faith is a positive factor in addiction prevention and that the value of faith-oriented approaches to substance abuse prevention and recovery is indisputable [49].

On the other hand, the current study found another set of variables that were not related to any individual, multiple or increasing ATOD behaviors. The two academic variables were not associated with ATOD use, in support of other studies [18,50,51]. Such lack of observed relationship might be explained by the indirect effects of substance use. For instance, alcohol might not lead to lower grades directly, but rather indirectly via impaired bodily/mental functions, where a path diagram showed that amount of alcohol consumed was associated with sleepiness and shorter sleep duration, which consequently affected students’ academic achievements [52]. 

Likewise, we found no associations between depressive symptoms and individual, multiple or increasing ATOD use, except for one isolated positive association between depression and one ATOD (IDU). Such general lack of associations agrees with others where despite 74.5% of university students reporting different degrees of depression, substance use as dysfunctional coping strategy had low scores on the Brief COPE questionnaire [53]. In agreement, given that among young adults, males cope with negative affects by using external avoidance-based coping, e.g., substances use, while females use e.g., rumination and isolation [54], hence, the 70% female composition of our sample and their significantly lower depressive score compared to males, might have collectively contributed to the observed general lack of detectable associations between depressive symptoms and any ATOD use in the current sample. Similarly, our observed lack of associations between stress and individual, multiple or increasing ATOD use concurs with research that employed the same tools as the current study, to report that stress was generally not associated with individual, multiple, or increasing ATOD use [11]. Nevertheless, studies found relationships between ATOD use on the one hand, and depressive symptoms and/or stress on the other. For instance, depressed mood was one of the best predictors of drug misuse among Puerto Rican students [55]; having higher depressive symptoms score was associated with ever IDU use among college students [56]; and undergraduates reported stress as a reason for psychoactive substance use [57]. Finally, income sufficiency was generally not associated with ATOD use, except for an isolated negative association between income sufficiency and one ATOD (smoking). 

This study has limitations. Data was collected at one university; and the sample remains a convenience sample. Cross sectional data do not infer causations. Data was self-reported; underreporting of ATOD cannot be ruled out. College populations are not essentially representative of young people in the country, so the generalizations should be cautious. The questionnaire did not ask about the time of initial use of illicit drugs which could have been useful to provide an overview of the time span during which respondent used such drugs. Our 27% response rate was based on the number of returned questionnaires in relation to the total number of students enrolled at the university. In agreement with others [45], it is not easy to calculate a precise response rate, as the research team did not have access to the exact numbers of students, who were reached by the mailing lists of the university (enrolled students who are not actively studying anymore, spam-filters, many students not using faculty mail services at all, etc.). Hence, our calculated response rate could be a cautious estimate, and the actual response rate may be higher. We allocated all three ATOD behaviors equal weighting; it might be argued that IDU could be assigned more weighting than tobacco smoking. Surveys among other student populations have been subject to similar limitations. Future research would benefit from addressing such limitations. Despite these limitations, the study has important strengths as to the best of our knowledge, this is the first study to appraise in detail, the prevalence and correlates of individual, multiple, and increasing ATOD use employing a wide range of socio-demographic, academic, and health and lifestyle characteristics across Finnish university students attending many different faculties.

## 5. Conclusions

Among this sample of college students, the prevalence of individual and multiple 3 ATOD behaviors is concerning, given the impact of ATOD. Individual ATOD use was significantly and considerably positively associated with the use of the other two substances. Multiple ATOD use was significantly negatively associated with being single or agreeing with total smoking or alcohol ban policy on campus; but significantly positively associated with not living with parents. Increasing ATOD use was significantly less likely among those in agreement with total smoking or alcohol ban policy on campus. Specific risk groups included single males not living with parents during semesters (and to a much lesser extent, good self-rated health, income sufficiency, depressive symptoms and religiosity) were associated to either individual, multiple or increasing ATOD behaviors. Thus, the findings of current study suggest that demographic variables seem more pertinent than lifestyle features in terms of ATOD use. Education and prevention efforts need to reduce risk, reinforce abstinence, and highlight the harmful outcomes. Health promotion interventions could be directed to explore students’ beliefs and expectations and target risk groups highlighted above. University strategies should be part of the wider country-wide successful ATOD control policies.

## Figures and Tables

**Table 1:** Descriptive characteristics of undergraduates by Gender (N = 1177).

Variable	Total N (%)	Female N (%)	Male N (%)	*p* *
	1177 (100)	823 (70.4)	346 (29.6)	
Socio-Demography				
Age years (M ± SD)	22.96 ± 5.21	23.01 ± 5.55	22.8 ± 4.36	0.548
Marital status				*0.001*
Married or in relationship	593 (50.7)	440 (53.9)	148 (42.8)	
Single	576 (49.3)	377 (46.1)	198 (57.2)	
Accommodation during semester				0.417
With parents	394 (33.7)	280 (34.1)	109 (31.7)	
Not with parents	776 (66.3)	540 (65.9)	235 (68.3)	
Income sufficiency				0.348
Always/mostly sufficient	488 (42.0)	348 (42.8)	136 (39.8)	
Always/mostly Insufficient	674 (58.0)	466 (57.2)	206 (60.2)	
Health				
Health awareness				*<0.001*
Not at all/not much	159 (13.6)	89 (10.9)	70 (20.4)	
To some extent/very much	1009 (86.4)	728 (89.1)	273 (79.6)	
Self-rated general health				*0.010*
Poor/Fair	87 (7.4)	50 (6.1)	36 (10.5)	
Good/very good/excellent	1083 (92.6)	768 (93.9)	308 (89.5)	
BDI Score (M ± SD)	50.88 ±18.4	52.73 ± 18.41	46.45 ± 17.90	*<0.001*
Perceived stress score (M ± SD)	14.14 ± 3.20	13.98 ± 3.21	14.52 ± 3.14	*0.009*
Lifestyle				
Smoking				0.108
Never	911 (78)	648 (79.2)	257 (74.9)	
Daily/Occasionally	257 (22.0)	170 (20.8)	86 (25.1)	
High frequency drinking (last 3 months)				*0.001*
No	691 (59.0)	507 (62.0)	179 (51.7)	
Yes	480 (41.0)	311 (38.0)	167 (48.3)	
Lifetime Illicit Drug/s Use				*0.001*
Never	921 (79.0)	669 (81.8)	249 (73.0)	
Ever	245 (21.0)	149 (18.2)	92 (27.0)	
Multiple ATOD behaviors				*0.001*
0 behavior	526 (45.6)	394 (48.8)	130 (38.5)	
1 behavior	362 (31.4)	254 (31.4)	104 (30.8)	
2 behaviors	188 (16.3)	111 (13.7)	77 (22.8)	
3 behaviors	77 (6.7)	49 (6.1)	27 (8.0)	
Religiosity (Importance of religion in life)				0.928
Low	702 (60.2)	493 (60.3)	206 (60.1)	
High	464 (39.8)	324 (39.7)	137 (39.9)	
Policy				
Total smoking ban on university premises				*0.001*
Strongly disagree/Disagree	233 (29.0)	138 (25.4)	94 (36.6)	
Strongly agree/Agree	571 (71.0)	405 (74.6)	163 (63.4)	
Total alcohol ban on university premises				*<0.001*
Strongly disagree/Disagree	208 (27.3)	125 (23.2)	81 (36.8)	
Strongly agree/Agree	554 (72.7)	413 (76.8)	139 (63.2)	
Academic				
Academic performance compared to peers				0.079
Same, better or much better	992 (84.6)	704 (85.9)	283 (81.8)	
Worse or much worse	180 (15.4)	116 (14.1)	63 (18.2)	
Importance of achieving good grades				*0.009*
Somewhat or very important	971 (83.1)	697 (85.0)	270 (78.7)	
Not important or not at all important	198 (16.9)	123 (15.0)	73 (21.3)	

Numbers in parenthesis represent column percentages unless otherwise indicated; * Two-sided *p*-values based on Pearson chi square test (categorical variables), and Student t test for comparison between means (continuous variables); M ± SD: Mean ± standard deviation; BDI: Beck Depression Inventory; High religiosity: Strongly or somewhat agree/neither agree nor disagree; Low religiosity: strongly disagree/somewhat disagree; Italics indicate statistical significance.

**Table 2:** Correlates of individual ATOD use by variables under examination across a sample of university students in Finland.

Variable	Daily/Occasional Smoking		High Frequency Drinking		Ever Illicit Drug Use	
	Adj OR (95% CI)	*p*	Adj OR (95% CI)	*p*	Adj OR (95% CI)	*p*
Gender (male)	1.046 (0.589; 1.857)	0.878	0.990 (0.620; 1.581)	0.967	2.463 (1.427; 4.252)	*0.001*
Age (years)	00.978 (0.928; 1.030)	0.403	1.011 (0.972; 1.052)	0.571	1.014 (0.965; 1.065)	0.578
Marital status (single)	0.661 (0.357; 1.225)	0.189	1.174 (0.707; 1.949)	0.536	0.332 (0.184; 0.600)	*<0.001*
Living during university (not living with parents)	1.959 (0.996; 3.855)	0.052	0.992 (0.586; 1.680)	0.976	3.253 (1.691; 6.258)	*<0.001*
Importance of religion (low: somewhat/strongly disagree)	1.370 (0.798; 2.350)	0.253	1.419 (0.928; 2.171)	0.106	1.551 (0.913; 2.633)	0.104
Income sufficiency (mostly/always sufficient)	0.581 (0.343; 0.986)	*0.044*	0.880 (0.572; 1.355)	0.563	0.862 (0.517; 1.439)	0.571
Self-rated general health (good/very good/excellent)	2.285 (0.792; 6.594)	0.126	1.250 (0.508; 3.079)	0.627	0.324 (0.134; 0.785)	*0.013*
Health awareness (to some extent/very much)	0.582 (0.293; 1.156)	0.122	1.895 (0.989; 3.633)	0.054	1.376 (0.671; 2.825)	0.384
Importance to achieve good grades (not very or at all important)	1.152 (0.580; 2.290)	0.685	0.713 (0.396; 1.282)	0.259	1.050 (0.540; 2.045)	0.885
Academic performance compared to peers (worse/much worse)	0.945 (0.442; 2.019)	0.884	1.285 (0.675; 2.447)	0.445	0.985 (0.478; 2.030)	0.967
Depressive symptoms score *^a^*	1.002 (0.982; 1.023)	0.836	0.988 (0.971; 1.005)	0.158	1.029 (1.009; 1.051)	*0.005*
Perceived stress score *^b^*	0.942 (0.842; 1.054)	0.299	1.012 (0.922; 1.111)	0.802	1.008 (0.903; 1.125)	0.893
Smoking ban on university premises (agree/strongly agree)	0.126 (0.072; 0.220)	*<0.001*	0.745 (0.440; 1.262)	0.274	0.491 (0.271; 0.891)	*0.019*
Alcohol ban sold on university premises (agree/strongly agree)	1.426 (0.763; 2.665)	0.267	0.136 (0.082; 0.226)	*<0.001*	0.894 (0.489; 1.637)	0.717
Smoking (daily/occasional)	—	—	2.654 (1.494; 4.716)	*0.001*	3.311 (1.833; 5.981)	*<0.001*
High frequency drinking (yes)	2.709 (1.534; 4.785)	*0.001*	—	—	1.963 (1.105; 3.486)	*0.021*
Ever illicit drug use (yes)	3.186 (1.767; 5.746)	*<0.001*	1.893 (1.072; 3.345)	*0.028*	—	—

Multiple logistic regression analyses; Adj OR: adjusted odds ratio, adjusted for all other variables in the table; CI: confidence interval; *^a^* Higher score = more depressive symptoms; *^b^* Higher score = more perceived stress; Italics indicate statistical significance.

**Table 3:** Correlates of multiple ATOD use across a sample of university students in Finland.

Variable	Multiple ATOD Use		
	Unstandardized β	95% CI	*p*
Gender (male)	0.123	−0.031; 0.277	0.118
Age (years)	0.001	−0.012; 0.015	0.862
Marital status (single)	−0.193	−0.357; −0.029	*0.021*
Living during university (not living with parents)	0.253	0.083; 0.423	*0.004*
Importance of religion (low: somewhat/strongly disagree)	0.126	−0.011; 0.264	0.072
Income sufficiency (mostly/always sufficient)	−0.092	−0.231; 0.046	0.191
Self-rated general health (good/very good/excellent)	−0.173	−0.450; 0.103	0.219
Health awareness (to some extent/very much)	0.098	−0.103; 0.299	0.337
Importance to achieve good grades (not very or at all important)	−0.057	−0.241; 0.127	0.542
Academic performance compared to peers (worse/much worse)	−0.014	−0.213; 0.185	0.890
Depressive symptoms score ^*a*^	0.003	−0.003; 0.008	0.327
Perceived stress score ^*b*^	0.003	−0.027; 0.033	0.836
Smoking ban policy on university premises (agree/strongly agree)	−0.743	−0.900; −0.587	*<0.001*
Alcohol ban policy on university premises (agree/strongly agree)	−0.467	−0.628; −0.307	*<0.001*

Multiple Linear regression analysis; CI: confidence interval; *^a^* Higher score = more depressive symptoms; *^b^* Higher score = more perceived stress; Italics indicate statistical significance.

**Table 4:** Correlates of increasing ATOD use across a sample of university students in Finland.

Variable	Increasing ATOD Use *^a^*					
	1 Risk Behavior versus No Risk Behavior		2 Risk Behaviors versus No Risk Behavior		3 Risk Behaviors versus No Risk Behavior	
	Adj OR (95% CI)	*p*	Adj OR (95% CI)	*p*	Adj OR (95% CI)	*p*
Gender (male)	1.080 (0.646; 1.805)	0.770	2.176 (1.087; 4.357)	*0.028*	1.451 (0.455; 4.629)	0.529
Age (years)	1.030 (0.989; 1.074)	0.154	0.957 (0.884; 1.036)	0.273	1.071 (0.960; 1.194)	0.219
Marital status (single)	0.590 (0.345; 1.009)	0.054	0.264 (0.119; 0.587)	*0.001*	0.536 (0.152; 1.890)	0.332
Living during university (not living with parents)	1.761 (1.010; 3.070)	*0.046*	4.333 (1.897; 9.897)	*0.001*	8.536 (1.880; 38.758)	*0.005*
Importance of religion (low: somewhat/ strongly disagree)	1.465 (0.936; 2.292)	0.095	2.893 (1.456; 5.748)	*0.002*	3.399 (1.115; 10.365)	*0.031*
Income sufficiency (mostly/always sufficient)	0.811 (0.512; 1.285)	0.373	0.508 (0.255; 1.011)	0.054	0.667 (0.232; 1. 919)	0.453
Self-rated general health (good/very good/excellent)	1.002 (0.382; 2.629)	0.997	0.528 (0.154; 1.815)	0.311	1.435 (0.241; 8.547)	0.691
Health awareness (to some extent/very much)	1.562 (0.762; 3.199)	0.223	0.789 (0.337; 1.844)	0.584	2.048 (0.477; 8.788)	0.335
Importance to achieve good grades (not very or at all important)	0.624 (0.326; 1.195)	0.154	0.865 (0.386; 1.936)	0.724	0.803 (0.183; 3.517)	0.770
Academic performance compared to peers (worse/much worse)	1.164 (0.582; 2.327)	0.668	1.341 (0.518; 3.473)	0.546	1.043 (0.249; 4.373)	0.955
Depressive symptoms score	1.002 (0.984; 1.020)	0.820	0.984 (0.960; 1.009)	0.211	1.029 (0.986; 1.073)	0.190
Perceived stress score	1.001 (0.905; 1.107)	0.981	0.960 (0.828; 1.113)	0.587	0.967 (0.786; 1.189)	0.748
Smoking ban policy on university premises (agree/strongly agree)	0.446 (0.247; 0.806)	*0.007*	0.165 (0.081; 0.335)	*<0.001*	0.037 (0.012; 0.116)	*<0.001*
Alcohol ban policy on university premises (agree/strongly agree)	0.216 (0.120; 0.388)	*<0.001*	0.159 (0.075; 0.338)	*<0.001*	0.243 (0.072; 0.827)	*0.024*

*^a^* Three separate multiple logistic regression models were used. Reference group is the ‘no risk behavior’ group; Adj OR: adjusted odds ratio, adjusted for all other variables in the table; italics indicate statistical significance.

## Data Availability

Data sharing not applicable.
